# Acute toxicity patterns and their management after moderate and ultra- hypofractionated radiotherapy for prostate cancer: A prospective cohort study

**DOI:** 10.1016/j.ctro.2024.100842

**Published:** 2024-08-17

**Authors:** F. Sinzabakira, L. Incrocci, K. de Vries, M.E.M.C. Christianen, M. Franckena, F.E. Froklage, H. Westerveld, W.D. Heemsbergen

**Affiliations:** a Department of Radiotherapy, Erasmus MC Cancer Institute, University Medical Center Rotterdam, Dr Molewaterplein 40, 3015 GD, Rotterdam, The Netherlands; b Department of Clinical Oncology, Rwanda Military Hospital, Street KK739TH, Kicukiro District, Kigali City, Rwanda

**Keywords:** Prostate cancer, Radiotherapy, Ultra hypofractionation, Moderate hypofractionation, Acute toxicity

## Abstract

**Objective:**

Hypofractionation has become the new clinical standard for prostate cancer. We investigated the management of acute toxicity in patients treated with moderate hypofractionation (MHF) or Ultrahypofractionation (UHF).

**Methods:**

In a prospective cohort setting, patients (N=316) received either MHF (20 fractions of 3/3.1 Gy, 5 fractions per week, N=156) or UHF (7 fractions of 6.1 Gy, 3 fractions per week, N=160) to the prostate +/- (base of the) seminal vesicles between 2019 and 2023. UHF was not indicated in case of significant lower urinary tract symptoms (LUTS) or T3b disease. Patient-reported outcomes (PRO) were online distributed at baseline, end of treatment (aiming at last fraction +/- 3 days), 3 months. Acute toxicity rates, management, and associations with baseline factors were analysed using Chi-square test and logistic regression. CTCAE scores (version 5) were calculated.

**Results:**

Treatment for acute urinary complaints was prescribed in 46 % (MHF) and 29 % (UHF). Taking into consideration baseline LUTS, MHF and UHF showed similar rates of PROs and management. Medication for acute gastrointestinal (GI) symptoms was prescribed for 21.1 % (MHF) and 14.1 % (UHF) with more loperamide for diarrhea in MHF (9.0 %) vs UHF (1.9 %, p = 0.005). Grade ≥ 2 (MHF / UHF) was scored in 40 % / 28 % for GI (p = 0.03) and 50 % / 31 % for GU (p < 0.01). PROs for GI reported after last fraction of UHF were significantly worse compared to before last fraction.

**Conclusion:**

UHF was safe with respect to acute toxicity risks in the selected population. MHF is associated with risks of significant diarrhea which needs further investigation. Furthermore, optimal registration of acute toxicity for UHF requires measurements up to 1–2 weeks after the last fraction.

## Highlights

• Hypofractionation has the potential to alleviate the burden on healthcare systems.
• In randomized trials ultra hypofractionation was feasible; data from clinical practice is limited.
• We report acute toxicity and management for moderate/ ultra hypofractionation in the clinic.
• Acute toxicity with moderate hypofractionation was as expected from previous trial results.
• Acute toxicity with ultra hypofractionation in selected patient group was acceptable as well.

## Introduction

External beam radiotherapy (EBRT) is an effective treatment modality for patients with localized prostate cancer. Traditionally, daily fractions of 1.8–2 Gy were administered, with the primary goal of optimizing tumour cell eradication while minimizing harm to adjacent healthy tissues [1]. Based on recent studies, the α/β ratio of prostate adenocarcinoma is considered low (around 1.5–3 Gy), which is different from most other tumors. Since the α/β ratio of surrounding tissue is assumed to be greater than that of the prostate tumor, treatment delivery with hypofractionated fraction sizes should theoretically improve the therapeutic ratio. In a number of randomized clinical trials it has been demonstrated that hypofractionation can be safely administered and is non-inferior to conventional fractionation in terms of efficacy and toxicity [2], [3], [4], [5], [6], [7], [8], [9]. Hypofractionation encompasses both moderate hypofractionation (MHF, 2.4–3.4 Gy per fraction) and ultrahypofractionation (UHF, ≥5 Gy per fraction) and has become the new clinical standard for prostate cancer [3].

The MHF schedule of the CHIIP trial delivers 60 Gy in 20 fractions (3 Gy per fraction) [4]. UHF offers even shorter treatment schedules and less required hospital visits compared to MHF, for instance the HYPO-RT-PC trial schedule of 42.7 Gy in 7 fractions [7], [10], [11], [12]. Amidst a global scarcity of radiotherapy services, researchers and clinicians have turned to hypofractionation to enhance accessibility and affordability [13], [14]. By decreasing the overall treatment cost and hospitalization duration, hypofractionation has the potential to alleviate the burden on healthcare systems [15].

Acute complications associated with radiotherapy typically reach their peak towards the end of treatment and are typically resolved three months post-radiotherapy [20]. The manifestation of bowel and urinary adverse events (e.g. irritative and obstructive urinary symptoms, diarrhea, cramping) require adequate management and treatment to optimize patient comfort and well-being [21]. Trials comparing UHF and MHF schedules with standard fractionation, indicate that HF might be associated with increased acute GI toxicity, however, the knowledge on the acute toxicity profiles and related management and the burden experienced by the patient by means of patient-reported outcomes, is currently limited [16]. The HYPO-RT-PC trial (UHF) and the HYPRO trial (MHF showed significantly increased patient-reported GI symptom rates compared to standard fractionation [17], [18], [19]. In the CHHIP trial (MHF) greater acute GI rates were observed as well, however, patient-reported outcomes were only collected for late endpoints [4], [20].

At the Erasmus MC Cancer institute, MHF (20 fractions of 3/3.1 Gy) was introduced in the clinic in 2019, and UHF (7 fractions of 6.1 Gy) in 2020, as a clinical protocol for our patient population with localized prostate cancer. In the current prospective cohort study we evaluated patient-reported acute toxicity and their management as a primary endpoint in the MHF and UHF cohorts. As a secondary endpoint, we reported acute toxicity according the Common Terminology Criteria for Adverse Events (CTCAE) scoring system.

## Methods and materials

### Patient population

**Fig. 1:**
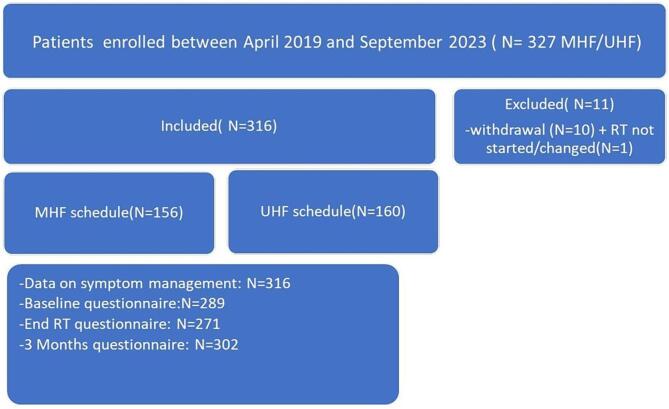
Flow chart of the study.

The PRORAD study is a prospective cohort study conducted at Erasmus MC Cancer Institute that includes prostate cancer patients receiving radiotherapy with curative intent according to one of the available clinical protocols (UHF, MHF, postoperative radiotherapy, brachytherapy, elective pelvic radiotherapy). Exclusion criteria were: previous pelvic radiotherapy, simultaneous treatment of other tumor, and not able to fill out Dutch questionnaires. The study protocol was reviewed and approved by the local METC (MEC 2018–1711, date of approval January 14, 2019). All study patients provided written Informed Consent. All procedures were performed in compliance with relevant laws and institutional guidelines. The study is registered at clinicaltrials.gov (NCT05645237). For the current study, N=316 patients treated with UHF or MHF within the PRORAD study cohort were eligible for analysis out of N=327 UHF and MHF patients who were included in the study between start in April 2019 and September 2023 (further details in Fig. 1 and Table 1).

**Table 1:** Baseline patients, tumour, and treatment characteristics. Bold values indicate statistical significant differences between UHF and MHF groups.

**Variables**	**Hypofractionation Group**		**P-value**
	**MHF (20X3/3.1 Gy, N=156)**	**UHF (7X6.1 Gy, N=160)**	
Age (Mean ± SD) years	73.4 (4.7)	72.8(5.4)	0.3
Age category 50–69	22.4 %	25.0 %	0.5
70–75	37.2 %	40.6 %	
76–85	40.4 %	34.4 %	
Previous TURP	19 (12.1 %)	12 (7.5 %)	0.2
Previous abdominal surgery	78 (50.0 %)	67 (41.8 %)	0.1
Diabetes Mellitus	34 (21.7 %)	25 (15.6 %)	0.1
T-stage			**<0.001**
T1c-T2a	44 (28.2 %)	93 (58.2 %)	
T2b-T2c	26 (16.6 %)	38 (23.7 %)	
T3a	26 (16.7 %)	29 (18.1 %)	
T3b (including n = 4 T4)	60 (38.5 %)		
Clinical target volume (median)	62.2 cm^3^	54.7 cm^3^	**0.001**
Gleason score			**<0.001**
6	16 (10.2 %)	25 (15.6 %)	
7	85 (54.4 %)	118 (73.7 %)	
8–10	55 (35.2 %)	17 (10.6 %)	
Androgen deprivation therapy 6 months prescription 12–18 months prescription 24–26 months prescription	95 (60.9 %) 20 (12.8 %) 68 (43.6 %) 7 (4.5 %)	30 (18.7 %) 7 (4.4 %) 22 13.8 %) 1 (0.6 %)	**<0.001**
Online replanning each fraction	−	40 (25.0 %)	
Seminal Vesicle (SV)- CTV			**<0.001**
Only base of SV	57 (36.5 %)	103 (64.3 %)	
Total SV	85 (54.4 %)	0 (0.0 %)	
Not included	12 (7.6 %)	56 (35.0 %)	
Baseline catheter	4(2.5 %)	0 (0.0 %)	**0.04**
LUTS			**<0.001**
IPSS>15 (regardless medication)	36 (23.1 %)	1 (0.6 %)	
Medication for LUTS at start RT	66 (42.3 %)	28 (17.5 %)	
IPSS>15 and/or medication	76 (48.7 %)	29 (18.1 %)	

Abreviations: SD=Standard Deviation; UHF=ultrahypofractionation; MHF=moderate hypofractionation; SV=Seminal Vesicles; IPSS=International prostate symptom score; LUTS=Lower Urinary tract symptoms; CTV=Clinical target volume.

### Diagnosis and treatment

For diagnosis, histological proven prostate cancer and a diagnostic MRI Scan was available for all patients (except in case of contraindications for MRI). In case of T3-T4 disease, Gleason ≥ 8, and/or PSA≥20, a PSMA scan (preferred) or bone scan was required as well. MHF was administered in 20 fractions with daily fractions of 3/3.1 Gy for 5 days per week (total dose 60/62 Gy). UHF was delivered in 7 fractions of 6.1 Gy per fraction to a total dose of 42.7 Gy (administered over 3 days per week for 2.5 weeks, including two weekends). UHF was introduced in the clinic in January 2020. Radiotherapy was administered using image-guided volumetric modulated arc radiotherapy (VMAT), with image guidance from internal prostate fiducial markers (for both MHF and UHF patients). Patients received full bladder and empty rectum instructions. Target volume included prostate +/- (base) seminal vesicles for all patients, with no elective radiation.

UHF was the preferred option because of its convenience and shorter Linac time. Patient were eligible for UHF in case of clinical cT1c-cT3a N0M0 disease and no presence of significant LUTS (according to protocol, IPSS score must be below 20, in daily practice, mainly patients with IPSS below 16 were included). Additional exclusion criteria were urinary incontinence, inflammatory bowel disease, no implanted markers, and a prostate volume > 90 cm^3^. In case of Gleason score ≥ 8 or cT3b, prescribed MHF dose was 62 Gy in 20 fractions; in all other cases MHF was delivered as 60 Gy in 20 fractions of 3 Gy. There was no policy of upfront prescription of alpha blockers at start of radiotherapy; at diagnosis, patients get a prescription for alpha blockers in case of significant LUTS, at the discretion of the treating physician (urologist or radiation oncologist).

The clinical target volume for cT1-cT3a cases was the prostate only in case of estimated low risk of SV invasion (PSA≤20 and Gleason score 6 or Gleason score 7 with an PSA≤4), in all other cT1-cT3a cases, the CTV consisted of prostate plus base (first 1 cm) of seminal vesicles (SV). For patients with high risk of SV involvement (PSA>20 and Gleason ≥ 8) and all T3b/T4 disease the total SV was included in the CTV. A margin of 5 mm (7 mm in caudal direction) was applied to the Clinical Target Volume (CTV) of prostate and base of the SV in case of UHF and prostate in case of MHF to generate a Planning Target Volume (PTV). For MHF a margin of 8 mm was applied for the (base of the) SV, when included in the CTV. Androgen deprivation therapy protocol was 18 months for T3-4 patients, and 6 months for T1-T2 patients with Gleason score ≥ 8. A diagnostic MRI was mandatory for UHF and preferred for MHF.

Applied dose constraints for rectum were volume ≥ 60 Gy < 1 %, volume ≥ 56 Gy < 15 %, volume ≥ 50 Gy < 30 %, (MHF), volume ≥ 42.7 Gy < 1 %, volume ≥ 38.4 Gy < 15 %, volume ≥ 28 Gy < 25 % (UHF). For small bowel and sigmoid: D2cc < 53 Gy (MHF), D2cc < 35 Gy (UHF). For bladder: volume ≥ 60 Gy < 5 % (MHF), and volume ≥ 42.7 Gy < 5 % (UHF). Dose constraints were partly based on proposed constraints of the CHHIP trial and HYPO-RT-PC trial [4], [7]. From September 2022 onwards, UHF patients were treated within an adaptive online protocol on a new installed linear accelerator with a high-quality cone beam CT and software for replanning of dose distributions prior to each fraction. In these cases, the actual position of the organs at risk was delineated prior to each fraction, and the same margins, dose constraints, and prostate matching procedure was applied.

### Patient-reported outcomes (PRO)

The study patients completed online questionnaires, providing information on acute toxicity at baseline, end of radiotherapy (RT), and 3 months post-RT. With respect to the “End RT” questionnaire, patients received an invitation around 3 days before their scheduled last fraction to fill out online the questionnaire within the coming week. Since treatment was sometimes rescheduled and deviating from the original planning, for some patients the timing of the “End RT” questionnaire was outside the planned time window. Questionnaires that were filled out after receiving at least 6 fractions (UHF) or 16 (MHF), up to 12 days after the last fraction were accepted for analysis (for this reason, n = 26 End RT questionnaires were excluded < 6/16 fractions, and n = 15 > too late). The patient was asked to score GU and GI complaints experienced in the preceding week (with response options no, a bit, quite a bit, very much), resembling the bowel and urinary domain of the EPIC (Expanded Prostate Cancer Index Composite) questionnaire and QLQ-PR25 Quality of Life questionnaire [22]. The EPIC and QLQ-PR25 are not suitable to measure acute toxicity because of the long recall time of 4 weeks, and because they do not specifically target acute toxicity. The questionnaire we used is based on items of the acute RTOG scoring system, with a recall time of one week. It has been previously used in several randomized trials to measure acute toxicity in radiotherapy for prostate cancer [17]. Further details on the PRO items are listed in Table 2, Table 3, Table 4. Results of the PRO are reported as “any complaint” and as “moderate to severe” (scores “quite a bit”, “very much”).

**Table 2:** Management (medication/interventions) of acute gastrointestinal and urinary adverse events.

**Acute adverse event management**	**Hypofractionation group**		**p value**
	**MHF (20x3/3.1)** **N=156**	**UHF (7X6.1)** **N=160**	
1. Treatment for GI complaints (doctor prescription)	33 (21.2 %)	23 (14.4 %)	0.1
1a. Medication for clinical constipation	6 (3.8 %)	8 (5.0 %)	0.6
1b. Psyllium/probiotica for stool consistency	9 (5.8 %)	6 (3.8 %)	0.3
1c. Medication for diarrhea (loperamide)	14 (9.0 %)	3 (1.9 %)	**0.005**
1d. Treatment for GI pain	10 (6.4 %)	9 (5.6 %)	0.8
−Treatment for anal pain (lidocaine oinment/pain killers)	7 (4.5 %)	5 (3.1 %)	0.7
−Medication for abdominal pain/cramps (mainly painkillers)	5 (3.2 %)	7 (4.4 %)	0.1
2. PRO: daily use of pads for uncontrolled loss (GI)	15 (9.6 %)	7 (4.4 %)	0.07
3. Treatment for urinary symptoms (doctor prescription)	71 (45.5 %)	47 (29.4 %)	**0.003**
3a. Catheter during RT (not at baseline)	11 (7.1 %)	4 (2.5 %)	0.09
3b. Medication for obstruction/frequency symptoms	60 (38.5 %)	43 (26.9 %)	**0.03**
3c. Pain medication for dysuria (all types)	12 (7.7 %)	8 (5.0 %)	0.3
4. PRO: daily use of pads for uncontrolled loss of urine	11 (7.1 %)	6 (3.8 %)	0.2
**Subgroup with IPSS≤15 and no LUTS medication at baseline**	**MHF (20x3/3.1)** **N=80**	**UHF (7X6.1)** **N=131**	**p value**
Treatment for urinary complaints	32 (40.0 %)	38 (29.0 %)	0.1
−Medication for obstruction/frequency complaints	28 (35.0 %)	36 (27.5 %)	0.2
−Catheter during RT (not at baseline)	4 (5.0 %)	2 (1.5 %)	0.3
**Subgroup with IPSS>15 or LUTS medication at baseline**	**MHF (20x3/3.1)** **N=76**	**UHF (7X6.1)** **N=29**	**p value**
Treatment for urinary complaints	39 (59.3 %)	9 (31.0 %)	0.1
− (Change in) medication for obstruction/frequency complaints	32 (42.1 %)	7 (24.1 %)	0.09
−Catheter during RT (not at baseline)	7 (9.2 %)	2 (6.9 %)	0.7

−Abreviations: RT=Radiotherapy; MHF=Moderate hypofractionation; UHF=Ultrahypofractionation; UTI=Urinary tract infection; GU=Genito-Urinary; GI=Gastro-intestinal, PRO=patient reported outcome.

−Bold values indicate statistical significance (*P*≤0.05).

**Table 3:** Patient-reported acute gastrointestinal adverse events at the end of radiotherapy. Calculated p value reflects Chisquare test for the distribution of none/any/moderate to severe. Bold values indicate statistical significant result (p<0.05).

**Acute Bowel** **adverse event**	**Base (N=289)** **Any/mod-s**	**MHF (N=133)** **Any/mod-s**	**UHF (N=138)** **Any/mod-s**	UHF * (N=64) **Any/mod-s**	**MHF vs UHF** **p value**	MHF vs UHF* p value
Painful defecation Abdominal cramps Urgency Mucus discharge Rectal bleeding Stool freq ≥ 4/≥6 Diarrhea	8.3 % / 1.0 % 9.3 %/2.0 % 15.5 %/1.3 % 4.1 %0.6 % 2.7 %/0.6 % 4.4 %/1.7 % 17.6 %/2.7 %	53.4 % / 19.5 % 62.4 %/24.8 % 63.4 %/30.1 % 62.4 %/24.8 % 31.6 %/6.0 % 50.4 %/24.8 % 69.2 %/21.8 %	34.8 % / 9.4 % 43.5 %/12.3 % 50.0 %/17.4 % 36.2 %/9.4 % 13.7 %/1.4 % 36.3 %/13.8 % 63.1 %/10.9 %	45.4 %/14.1 % 54.7 %/17.2 % 57.8 %/23.4 % 50.0 %/15.6 % 18.8 %/1.6 % 56.3 %/18.8 % 76.5 %/15.6 %	**0.005** **0.003** **0.004** **<0.001** **0.002** **0.03** **0.04**	0.5 0.4 0.3 0.1 0.1 0.2 0.2

Abbreviations: MHF=moderate hypofractionation; UHF=ultrahypofractionation, mod-s = moderate to severe.

UHF*: Questionnaire at least 13 days after the first fraction.

**Table 4:** Patient-reported acute urinary adverse events at the end of Radiotherapy. Calculated p value reflects Chi square test for the distribution of none/any/moderate to severe. Bold values indicate statistical significant result (p<0.05).

**Acute urinary** **adverse event**	**Base (N=289)** **Any/moderate-severe**	**MHF (N=133)** **Any/moderate-severe**	**UHF (N=138)** **Any/moderate-severe**	**MHF vs UHF** **p value**
Painful urination	12.9 %/1.4 %	69.4 %/30.6 %	62.7 %/23.1 %	0.3
Straining	24.2 %/4.2 %	76.1 %/33.1 %	72.4 %/28.4 %	0.6
Hematuria	4.2 %/0.7 %	0.8 %/0.0 %	2.9 %/0.7 %	0.4
Nocturia ≥ 5/night	2.8 %	31.4 %	19.4 %	**0.02**
**IPSS <15 and no LUTS medication**	**Base (N=198)** **Any/moderate-severe**	**MHF (N=73)** **Any/moderate-severe**	**UHF (N=111)** **Any/moderate-severe**	**MHF vs UHF** **p value**
Painful urination	9.5 %/1.1 %	72.6 %/30.1 %	63.9 %/21.6 %	0.1
Straining	15.8 %/1.1 %	78.0 %/32.5 %	72.0 %/28.8 %	0.3
Hematuria	5.3 %/1.1 %	0.0 %/0.0 %	3.8 %/0.9 %	0.1
Nocturia ≥ 5/night	1.1 %	25.0 %	20.0 %	0.4
**IPSS>15 or LUTS medication at baseline**	**Base** **Any/moderate-severe**	**MHF** **Any/moderate-severe**	**UHF** **Any/moderate-severe**	**MHF vs UHF** **p value**
Straining	41.0 %/10.5 %	73.7 %/35.1 %	75 %/29.2 %	0.5
Nocturia ≥ 5/night	6.4 %	38.6 %	16.7 %	0.7

Abbreviations: MHF=moderate hypofractionation; UHF=ultrahypofractionation.

### Assessments and CTCAE scoring

Patients had standard assessments once during their course of treatment in case of UHF and twice in case of MHF. In addition, patients were seen in case of symptoms that need attention, upon request of the patient or the technicians who see the patient at each fraction. All patients were assessed 3-months post-RT and available documentation of other hospitals was reviewed as well. For scoring the acute toxicity according to CTCAE version 5, we considered all system organ class items of the categories “gastrointestinal disorders” and “renal and urinary disorders”.

### Statistical analysis

Statistical analyses were conducted using IBM SPSS version 25 (IBM Corp, Armonk, New York). Pearson Chi-square tests were used to compare categorical variables. Symptom management percentages were calculated for total groups and for subgroups based on baseline symptoms. Univariable binary logistic regression analysis was performed to evaluate associations between acute toxicity endpoints and baseline parameters. All statistical tests were two-sided, with a significance level set at p < 0.05.

## Results

### Baseline characteristics

Since UHF patients were selected based on T stage, baseline urinary symptoms, and limited prostate volume, several treatment-related characteristics were significantly different between the UHF (N=160) and MHF (N=156) group, like prescription of androgen deprivation therapy, baseline LUTS medication, clinical target volume, and T stage distributions (Table 1). Within the UHF group, N=40 patients received adaptive treatment. With respect to previous TURp, 6.4 % of the MHF group had a TURp in the preceeding 12 months and 5.8 % >12 months before RT; for UHF these numbers were 1.9 % and 5.6 %, respectively. Nine patients with ADT indication did not receive ADT (n = 5 because of cardiovascular comorbidity, n = 3 because of patient preference, n = 1 because of high age). Ten patients with ADT indication of 6 months according protocol because of Gleason score ≥ 8 and T1-T2, had ADT prescription of 12–18 months. Nine patients with a T3a staging for the radiotherapy plan based on imaging but with a clinical T1c tumor (digital rectal exam), had no ADT prescription.

### Acute toxicity management

With respect to management of GU toxicity, no clinically relevant differences between UHF and MHF were observed, taking into account differences in baseline LUTS (Table 2). A total of 45.5 % in the MHF group and 29.3 % in the UHF group needed treatment (medication or catheter) for acute urinary toxicity. With respect to GI toxicity, these numbers were 21.1 % and 14.4 %, respectively. Regarding the number of patients treated for diarrhea (with loperamide), we observed significant differences between the MHF and UHF cohort (9.0 % MHF vs. 1.9 % UHF, p = 0.005) (Table 2). Three UHF patients received temporary opioid medication for severe GI pain: two patients used oxycodone prescribed by the radiation oncologist, because of severe anal pain, and one patient used morphine prescribed by the emergency room because of severe pain with defecation and urination.

### Patient-reported outcomes

**Fig. 2:**
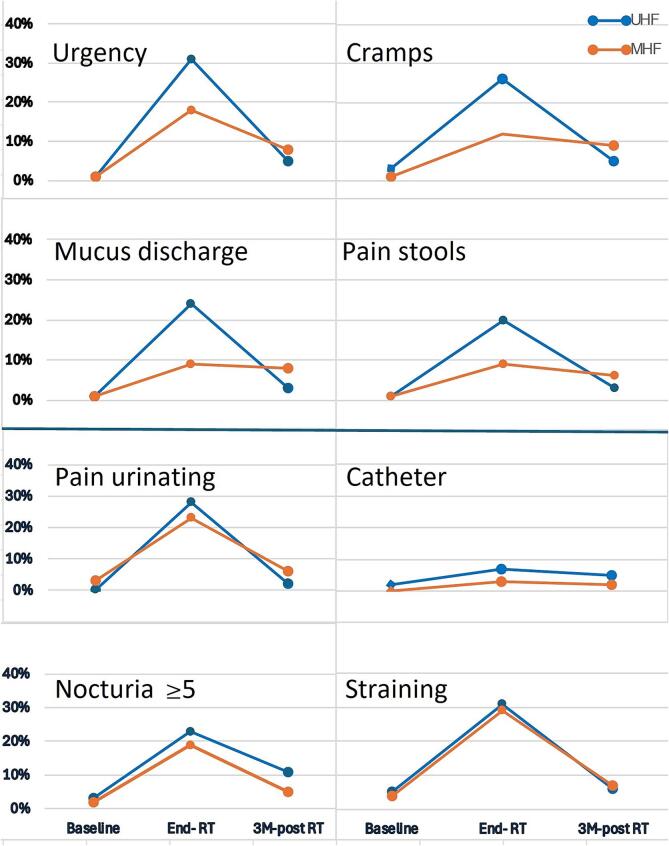
The incidence of patient-reported acute bowel and urinary complaints over time (moderate to severe), with percentage of patients reporting the indicated complaints (Y-axis) at each measured phase of the acute toxicity period (X-axis).

At the end of radiotherapy, patients treated with MHF experienced statistically significant more often GI toxicity compared to the UHF group (Table 3). For UHF patients however, a correlation between symptom rate and questionnaire interval was observed: UHF patients who completed the acute questionnaire ≥ 13 days between first fraction and the questionnaire “End RT” reported more acute GI side effects, therefore we added separately the PRO toxicity rates for this subgroup (N=64) in Table 3. The trajectory of bowel complaints over time is depicted in Fig. 1 (moderate to severe) and Supplementary Figure A1 (any complaint). As expected, by 3 months post-irradiation, toxicity rates had regressed toward baseline levels in both groups. With respect to patient-reported acute urinary toxicity (Table 4), similar levels of adverse events were observed for MHF and UHF, when considering the baseline LUTS. The trajectory of urinary adverse events over time is depicted graphically in Fig. 2.

### Common Terminology criteria for adverse event (CTCAE) scores

According CTCAE version 5, adverse events requiring medical management, temporary catheters, daily pads, and/or cause moderate pain, and stool frequency increase over baseline ≥ 4–6, were scored as Grade 2. A stool frequency increase over baseline ≥ 7 and severe pain were scored as Grade 3. As a result, grade ≥ 2 (MHF / UHF) was scored in 40 % / 28 % for GI and 50 % / 31 % for GU, with p = 0.03 and p < 0.01, respectively. Grade 3 GI was scored in 7 % of MHF cases and 9 % of UHF cases, mainly based on increased stool frequency ≥ 7 over baseline. No Grade 3 GU cases were scored.

### Correlation between acute toxicity and baseline variables

Supplementary Table A1 shows the associations between baseline characteristics and acute toxicity outcomes. We found that baseline complaints (typically being mild at that time) was significantly associated with worsening to moderate-severe. We further analysed the impact of baseline LUTS/medication on the risk of developing severe LUTS during the acute period (defined as a catheter or nocturia ≥ 7) in UHF and MHF subcohorts, and a strong correlation was found, with a 3.5 % risk for IPSS category 0–7 (without medication) up to a 21 % risk for the IPSS category > 15/LUTS medication, as shown in Supplementary Table A2.

## Discussion

We conducted a prospective study on incidence and management of acute toxicity after modern EBRT with MHF or UHF for patients with localized prostate cancer. In general, baseline LUTS was associated with a higher risk of needing a transurethral catheter for acute urinary retention. The outcomes suggested similar acute GU toxicity profiles for UHF and MHF, considering the difference in inclusion criteria with respect to baseline IPSS. MHF was associated with significantly more prescription of anti-diarrheal medication. This corresponded with a significantly higher rate of patient-reported GI complaints (including diarrhea) at the end of radiotherapy for MHF vs UHF. However, GI toxicity reported by UHF patients were significantly more frequent when the questionnaire was filled out around the last fraction or later, which indicates that for some UHF patients (with earlier questionnaires) we might have missed the peak of clinical adverse events.

In a review by Andreyev et al., it is pointed out that the acute damage in the rectal mucosa typically develops during the second week of radiation whereas the clinical symptoms typically appear in the weeks thereafter when histological changes are stable or improving, peaking in week 4–5, which is based on 6–7 weeks of treatment with 2 Gy fractionation [21]. This phenomenon explains why UHF patients in our cohort who filled out PROs within 2 weeks, reported less complaints. Therefore, in the specific situation of UHF with short overall treatment times, it is essential that measurements of acute toxicity include at least the third week after start treatment.

Comparing the biologically effective dose (BED) of the different dose-fractionation schedules (Supplementary Table A3), with respect to the prostate (with an assumed low α/β of 1.5–3 Gy) and acute tissue response (α/β = 10 Gy) we find that in theory the 7x6.1 Gy schedule has the most favourable therapeutic ratio with a tumor BED of 130–216 Gy (versus 120/127–180/190 Gy for 20x3/3.1 Gy) and an acute tissue BED of 69 Gy (versus 78/81 Gy). However, looking at the dose rate per week (since time is also a factor for acute toxicity), the 7x6.1 Gy has the highest BED rate per week for acute toxicity. Whether the BED for late toxicity is in theory also more favourable for 7x6.1 Gy compared to 20x3/3.1 Gy, depends on the exact α/β ratio of the late tissue.

The general picture of the large clinical trials evaluating HF schedules versus CF schedules with respect to acute toxicity, is that urinary complaints show comparable or slightly increased levels between UHF/MHF and CF, and bowel toxicity show higher levels [7], [8], [12], [17], [20], [18]. In the CHHIP trial, reported acute Grade ≥ 2 toxicity rates (RTOG scores) in the 20x3 Gy MHF arm were 38 % for GI and 49 % for GU, which is almost identical to the numbers for MHF in our study (40 % and 50 %, respectively, using the CTCAE scoring system). The Scandinavian HYPO-RT-PC trial [18], which compared UHF (7X6.1 Gy) versus CF (39x2 Gy), revealed significantly higher levels of acute patient-reported bowel toxicity in the UHF group compared with the SF group [12], [18]. Significant clinically relevant deteriorations were observed for increased stool frequency, urgency, flatulence, abdominal cramp, mucus discharge, and blood in stools [18]. With respect to physician-based acute toxicity scores, they reported no significant differences. In general, this in line with our results since we also observed relatively high acute patient-reported toxicity rates, for both UHF and MHF, compared to the previous clinical standard of 78 Gy in 2 Gy fractions. Acute toxicity was measured in this trial “at end of treatment”, which suggests that there might have been patients in their study as well who developed clinical symptoms after treatment which were not scored.

In the PACE-B trial patients were randomized between an experimental arm of UHF (5X7.25 Gy) and a combined standard arm of MHF (20x3.1 Gy), which was received by 70 % of this arm, or CF (39 x 2 Gy) [13]. They reported that UHF did not show significantly worse acute toxicity compared to the standard arm of mainly MHF patients. These results are roughly in agreement with our current results.

Research into acute toxicity management strategies during prostate cancer radiotherapy, particularly concerning the recently clinical introduced UHF and MHF treatment regimens, are scarce. Dahiya et al. reported on medical management strategies for acute and late radiation proctitis. They concluded that acute proctitis is for most cases self-limiting and treatment of patients with acute RP is usually supportive, consisting of hydration, anti-diarrheal agents, and fibres [23]. In the HYPRO trial a limited set of management items for acute toxicity was reported with respect to medication for pain (GI, GU), medication for diarrhea, and use of pads [20]. In a review paper of Pancy et al., management and strategies for (sub)acute and late LUTS after RT in general is discussed in detail, including lifestyle advice for patients [24].

A subgroup of UHF patients was treated with adaptive radiotherapy (ART) to optimize the dose distribution to the actual position of the organs at risk prior to each fraction (bladder, rectum, sigmoid/small bowel). This was performed using the same margins and the same dose constraints as applied for the standard UHF planning on a planning CT scan. Literature on ART for PCa patients is mainly based on MR-Linac studies, and show reduced toxicity when applying smaller margins as part of such a strategy. However, additional clinical benefits of ART only have not been demonstrated yet with inconclusive results so far [25], [26], [27]. We have planned to analyse the potential benefits of this adaptive strategy when we have reached a minimum sample size of 80 UHF patients and have collected all relevant dose data to perform a meaningful analysis.

An important limitation of the current study is the unbalanced patient allocation to MHF/UHF with respect to baseline LUTS and adaptive strategies, hindering an unbiased comparison of UHF and MHF. The lack of a detailed management protocol for treating complaints might have affected results as well to some extent, there was however a general consensus and regular exchange of opinions between radiation oncologists. A strong point of our study is its prospective nature and the setting of an unselected patient population with respect to comorbidity and age.

In conclusion, UHF was safe with respect to acute toxicity risks in the selected patient population. Assessment of the benefits of an online adaptive strategy for UHF is needed to establish its cost-effectiveness. MHF showed similar acute toxicity profiles as reported in the CHHIP trial. An evaluation of late toxicity and tumor control is planned for the near future, to complete the evaluation of UHF and MHF protocols for daily clinical practice.

## Declaration of Competing Interest

The authors declare that they have no known competing financial interests or personal relationships that could have appeared to influence the work reported in this paper.
