# Supplementary material for: Acute toxicity patterns and their management after moderate and ultra- hypofractionated radiotherapy for prostate cancer: A prospective cohort study
Source: Clin Transl Radiat Oncol. 2024 Aug 17;48:100842. doi: 10.1016/j.ctro.2024.100842 (PMC11387742; doi:10.1016/j.ctro.2024.100842)
Supplement: Supplementary Data 1 [file mmc1.docx]

**Appendix A. Supplementary Files Sinzabakira et al.**

**Figure A1.** The incidence of patient-reported acute bowel complaints over time (any complaint), with percentage of patients reporting the indicated symptoms (Y-axis) as a function of time (X-axis)


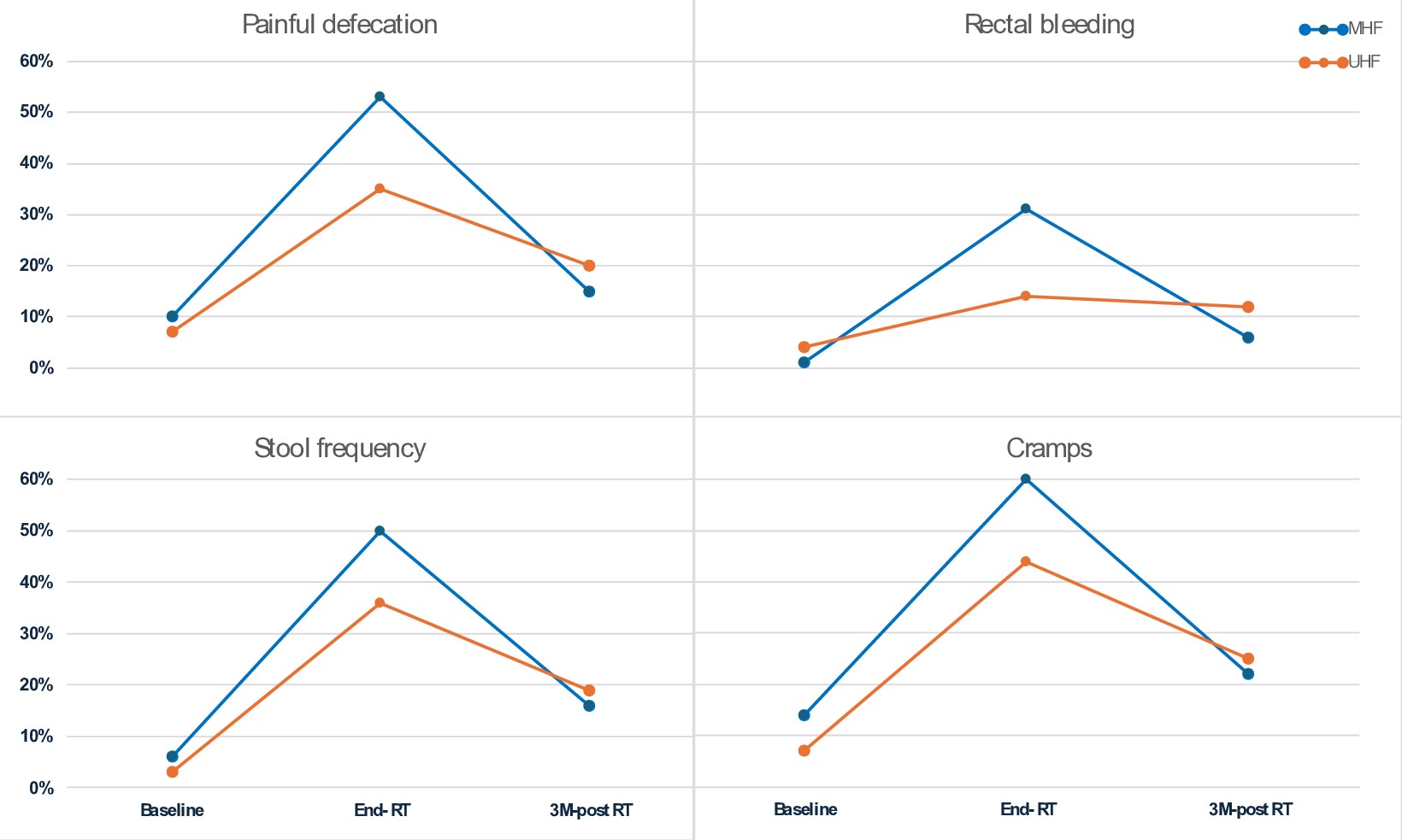


**Table A1.** Association between baseline parameters and most frequent moderate to severe patient-reported acute symptoms, and GI and GU management endpoints. Results from univariable logistic regression models.

| **Baseline features** | **PATIENT-REPORTED SYMPTOMS (moderate-severe)** | | | | | | **MANAGEMENT** | |
| --- | --- | --- | --- | --- | --- | --- | --- | --- |
|  | **Straining** | **Urinary Pain** | **Diarrhea** | **GI Urgency** | **Cramps** | **Stool freq**  **≥4** | **GI treatment** | **GU treatment** |
|  | **OR, p** | **OR, p** | **OR, p** | **OR, p** | **OR, p** | **OR, p** | **OR, p** | **OR, p** |
| Age>70 vs <=70  Abd surgery yes vs no  Diabetes yes vs no  AHT yes vs no  IPSS >=15 vs <15  T3/4 vs T1/T2  CTV >60 cm^3^ vs <60  Baseline symptom | 1.05, 0.8  1.07, 0.8  1.5, 0.2  0.7, 0.2  1.12, 0.6  0.8, 0.5  1.5, 0.18  1.4, <**0.01** | 0.6, 0.1  1.4, 0.1  1.3, 0.3  0.6, 0.1  1,3, 0.3  0.6, 0.1  1.8, 0.06  1.5, <**0.01** | 1.4, 0.3  1.09, 0.7  0.8, 0.7  0.9, 0.9  1.2, 0.5  1.1, 0.7  1.3, 0.4  1.5, <**0.01** | 1.3, 0.3  0.8, 0.7  1.1, 0.6  1.2, 0.4  1.8, **0.04**  1.2, 0.5  1.6, 0.16  1.4, <**0.01** | 1.1, 0.6  0.9, 0.7  2, 0.5  1.2, 0.4  1.1, 0.7  1.1, 0.6  1.2, 0.6  1.4, **0.01** | 0.7, 0.4  0.8, 0.5  1.4, 0.3  1.2, 0.4  1.5, 0.1  1.3, 0.3  1.4, 0.2  1.3, 0.1 | 1.3, 0.2  0.6, 0.1  1.08, 0.8  1.2, 0.5  1.01, 0.9  1.3, 0.2  1.3, 0.3 | 1.3, 0.2  1.1, 0.5  1.6, 0.07  1.2, 0.3  1.1, 0.1  1.3, 0.2  1.1, 0.8 |
| Abbreviations: OR= Odds ratio; Freq=Frequency; GI=Gastrointestinal; GU= Genitourinary; CTV=clinical target volume  Bold p values represent significancy (p˂0.05) | | | | | | | | |

**Table A2.** Endpoint of severe LUTS in the acute phase of radiotherapy (catheter during radiotherapy course or nocturia ≥7 (N=312 pt with no catheter at baseline).

| **Hypofractionation Group** | **Baseline IPSS Category** | **Catheter or nocturia ≥7 (n=33)** | **p** |
| --- | --- | --- | --- |
| MHF (N=152) | 0-7 | 3/51 (5.9%) | 0.005 |
|  | 8-15 | 4/28 (14.3%) |  |
|  | ˃15 or LUTS medication | 18/73 (24.7%) |  |
| UHF (N=160) | 0-7 | 2/91 (2.2%) | 0.05 |
|  | 8-15 | 3/35 (7.9%) |  |
|  | ˃15 or LUTS medication | 3/26 (10.3%) |  |
| Total group (N=312) | 0-7 | 5/139 (3.5%) | ˂0.001 |
|  | 8-15 | 7/59 (10.6%) |  |
|  | ˃15 or LUTS medication | 21/81 (20.6%) |  |

**Table A3.** Calculated BED for different dose-fractionation schedules and different α/β ratios (currently applied hypofractionation schedules in the clinic, and the previous clinical standard schedule of 78 Gy in 2 Gy fractions).

| **Treatment schedule** | **Physical dose** | **α/β = 1.5** | **α/β = 3** | **α/β = 5** | **α/β =10** |
| --- | --- | --- | --- | --- | --- |
| ***Total treatment*** |  |  |  |  |  |
| 39x2 Gy (5x per week) | 78 Gy | 182 Gy | 130 Gy | 109 Gy | 93.6 Gy |
| 20x3 Gy (5x per week) | 60 Gy | 180 Gy | 120 Gy | 96.0 Gy | 78.0 Gy |
| 20x3.1 Gy (5x per week) | 62 Gy | 190 Gy | 127 Gy | 100 Gy | 81.2 Gy |
| 7x6.1 Gy (3x per week) | 42.7 Gy | 216 Gy | 130 Gy | 94.8 Gy | 68.8 Gy |
| ***Dose rate per week*** |  |  |  |  |  |
| 5x2 Gy | 10 Gy | 23.3 Gy | 16.7 Gy | 14.0 Gy | 12.0 Gy |
| 5x3 Gy | 15 Gy | 45.0 Gy | 30.0 Gy | 24.0 Gy | 19.5 Gy |
| 5x3.1 Gy | 15.5 Gy | 47.5 Gy | 31.5 Gy | 25.1 Gy | 20.3 Gy |
| 7x6.1 Gy | 18.3 Gy | 92.7 Gy | 55.5 Gy | 40.6 Gy | 29.5 Gy |
